# Validation of the Patient Neurotoxicity Questionnaire for Patients Suffering From Chemotherapy-Induced Peripheral Neuropathy in Greek

**DOI:** 10.7759/cureus.14324

**Published:** 2021-04-06

**Authors:** Theofilos Tsoleridis, Pelagia Chloropoulou, Athanasia Tsaroucha, Athina Vadalouca, Ioanna Siafaka, Theodosia Vogiatzaki

**Affiliations:** 1 Anesthesia and Pain Treatment Unit, General Hospital of Rhodes, Rhodes, GRC; 2 Department of Anesthesiology, Democritus University of Thrace, Alexandroupolis, GRC; 3 First Department of Anesthesiology, Pain and Palliative Care, University of Athens Medical School, Athens, GRC; 4 Pain and Palliative Care Center, Athens Medical Hospital, Athens, GRC

**Keywords:** chemotherapy, pain, peripheral neuropathy, validation, questionnaire

## Abstract

Purpose: The Patient Neurotoxicity Questionnaire (PNQ) represents a diagnostic tool concerning patients with chemotherapy-induced peripheral neuropathy (CIPN). The application of such a tool in the Greek clinical praxis requires validation.

Methods: Validation consists of three stages - translation, reverse translation, and patient application. Hundred oncologic patients were assessed by comparing the PNQ to the National Cancer Institute-Common Terminology Criteria for Adverse Events (NCI-CTCAE) at the chemotherapy onset and second, fourth, and sixth sessions. The diagnostic tool's specific requirements (compliance, validity, concordance, sensitivity, specificity, reliability) were statistically evaluated.

Results: Differences between translated texts and between the reverse translation and the original were considered negligible. At the second, fourth, and sixth session compliance was 98%, 95%, and 93% while Cronbach’s α was 0,57 0,69, and 0,81, respectively. Cohen’s weighted κ was 0,67 and 0,58, Spearman’s ρ was 0,7 and 0,98, while the area under the curve (AUC) of the receiver operating characteristic (ROC) was 1 and 0,9 for the sensory and the motor part, respectively. The variance’s linear regression analysis confirmed CIPN worsening over time (P<0.0001).

Discussion: The Greek version remains close to the original English version. Compliance rates reflect easy PNQ applications. Cohen’s κ values highlight the physicians’ tension to underestimate the patients’ condition. Spearman’s ρ, Cronbach’s α, and AUC values reflect good validity, reliability, and specificity of the PNQ respectively. Finally, the linear analysis confirmed the PNQ sensitivity over time.

Conclusions: The PNQ validation in Greek adds a crucial tool to the physicians' armamentarium. It can now delineate the necessary information to modify the chemotherapy and analgesic treatment regimens at both preventive and acute levels.

## Introduction

Chemotherapy-induced peripheral neuropathy (CIPN) represents an important issue for patients and is considered to have a devastating impact on the patients’ quality of life. Plus, there is no specific treatment or prevention. CIPN symptomatology is subjective, and for that reason, diagnosis and management of CIPN are hindered due to a lack of reliable and standardized methods [1-4].

The use of diagnostic tools for CIPN assessment has to be both practical and comfortable, not only for the patients but also for physicians. It should not be time-consuming, either interventional, and has to fulfill specific features. One such method that complies with the aforementioned criteria and is increasingly used in clinical praxis with significantly positive results, is the Patient Neurotoxicity Questionnaire (PNQ). The PNQ represents a simple self-administered assessment tool created by BioNumerik Pharmaceuticals, Inc. containing data from the FDA. It involves specific questions that aim to draw quantifiable and important diagnostic information directly from CIPN patients that regard both the severity and incidence of subjective CIPN symptoms [1].

The element that propelled the urge for the creation of PNQ was the weakness of other questionnaires to assess and quantify CIPN reliably. Despite that CIPN is often reported in many studies, the frequency, prevalence, onset, duration, regression, or persistence of CIPN in patients that undergo chemotherapy are unknown. Furthermore, the number of patients that face treatment delay or modification because of CIPN is rarely reported in clinical studies. Recent studies regarding CIPN symptomatology that compare patient-centered reporting methods to physician-centered reporting methods prove that physicians tend to under-report the severity and frequency of CIPN in comparison to the patients [5-12].

In order to apply such a tool in the Greek daily clinical praxis, a validation procedure is needed. That way, the contents of the questionnaire can be fully understood by the population, and useful information can be extracted [5,6].This study regards the validation procedure of the PNQ in Greece after obtaining the permission of the original author and the analysis of the data collected from the patients.

## Materials and methods

The linguistic process consists of three stages. The first stage concerns the translation of the questionnaire to the target language by two professional translators that perform the task separately. Then, the two translated texts are compared to ascertain if they are identical or not. In the second case, the nature of the differences has to be clarified (conception, comprehension, synonyms) and countered to conclude into an identical text or to a text that contains both translation versions (if needed) [5,6].

The second stage regards the reverse translation of the questionnaire from the target language to the original by a third professional translator. The result is again compared to the original to ascertain that they are identical or -if different- to examine the nature of the differences. In case of substantial differences, the resulted text of the first stage has to be reassessed, and if that is not possible, the procedure is repeated [5,6].

Finally, the third stage consists of the application of the translated questionnaire on patients. In similar studies that regarded the validation of cancer-specific questionnaires such as the European Organisation for Research and Treatment of Cancer Quality of life Questionnaire (EORTC QOL-Core 30) and the Functional Assessment of Cancer Therapy/Gynecologic Oncology Group-Neurotoxicity (FACT/GOG-Ntx) in the United States (two studies) and that of the Quality of Life-Anti-Cancer-Drugs (QOL-ACD) in Japan involved 305, 545, 262, and 212 patients, respectively [5-11].

Considering that the General Hospital of Rhodes receives about 600 patients for chemotherapy every year and the significant difference in the population between Greece, and Japan, and the USA, a sample of 100 patients (with 95% confidence level and 8.9 confidence interval), was considered enough for validating the PNQ in Greek. The entry criteria were adult male or female patients under 80 years of age that would undergo chemotherapy for the first time, regardless of cancer type, social and educational underground, without pre-existing neurologic condition, and able to communicate with personnel. Therefore, patients with a pre-existing neurological condition or already under chemotherapy treatment or unable to communicate with personnel as well as pediatric patients were excluded from the study.

The study was executed from April 2019 to October 2020 in oncologic patients of the General Hospital of Rhodes, Greece, after the approval of the Ethics Committee of the hospital (protocol number 6511, protocol registration and results system of the ClinicalTrials.gov database {PRS} number NCT04773379). The patients were informed about the contents of the questionnaire and the benefits of validating and developing an assessment tool that would help both analgesic and chemotherapeutic regimens. After their informed consent, the patients alone compiled the questionnaires anonymously, without physician-patient, nurse-patient, or patient-patient conversation or consultation. CIPN assessment was based on comparing the patient-centered PNQ to the physician-centered National Cancer Institute-Common Terminology Criteria for Adverse Events (NCI-CTCAE). Both questionnaires were compiled at the chemotherapy onset and the second (up to one month after treatment onset), fourth (up to two months after the second session), and sixth (up to two months after the fourth session) treatment sessions.

The present study involved a comparison between PNQ and the NCI-CTCAE based on symptomatology importance. This importance is graded at the PNQ from A (no symptoms) to E (maximum symptomatology), while at the NCI-CTCAE is graded from 0 (no signs) to 4 (maximum symptomatology).

As mentioned above, a diagnostic assessment tool has to satisfy some specific requirements. These include compliance, which is the percentage of valid questionnaires compiled at each treatment session, and validity, the diagnostic tool's ability to comply with specific conditions to produce acceptable results. The latter can be measured using linear correlation models such as Spearman's ρ correlation coefficient [1,7,12-16].

Concordance is another requirement and represents the degree of correlation of the diagnostic tool with the detection of the condition's symptomatology's progress over time and treatment dosage. Concordance can be calculated by measuring the agreement rate between two different tools and can be achieved by calculating Cohen's κ coefficient [6,13-16]. To assess the responsiveness and sensitivity of the PNQ, again, the nominal grading was converted to numeral, and weight coefficients were distributed for each session [6,9,15].

Like the sensitivity of a diagnostic tool, responsiveness reflects the ability to distinguish positive or negative changes over time. This can be calculated by using linear regression models of variance. As chemotherapy results are additive, the registry of continuing higher scores is expected in each treatment session.

The reliability of a diagnostic tool reflects the ability to produce the right results for a specific period regardless of conditions and can be measured by calculating Cronbach's α coefficient. To calculate Cronbach's α, the nominal grading of the PNQ (A-E) was again converted into numeral grading (0-4). Finally, the Specificity of a diagnostic tool, which is the ability to register flawless calculations, can be assessed by analyzing the receiver operating characteristic curve (ROC curve) depending on sensitivity [8,17-21].

The ROC curve represents a tool that can assess a binary distribution system's diagnostic ability and is composed of a true positive rate axis and a false positive rate axis. The latter is equal to 1 - sensitivity. As mentioned above, the data were distributed in a binary manner. Patients with no neuropathy consisted of the "0" group, while those with neuropathy consisted of the "1" group. Again the nominal grading of the PNQ was converted to numeral, with the patients presenting a score >1 being distributed to the "1" group. The same was applied to the NCI-CTCAE [6,8,15,16].

## Results

**Figure 1:**
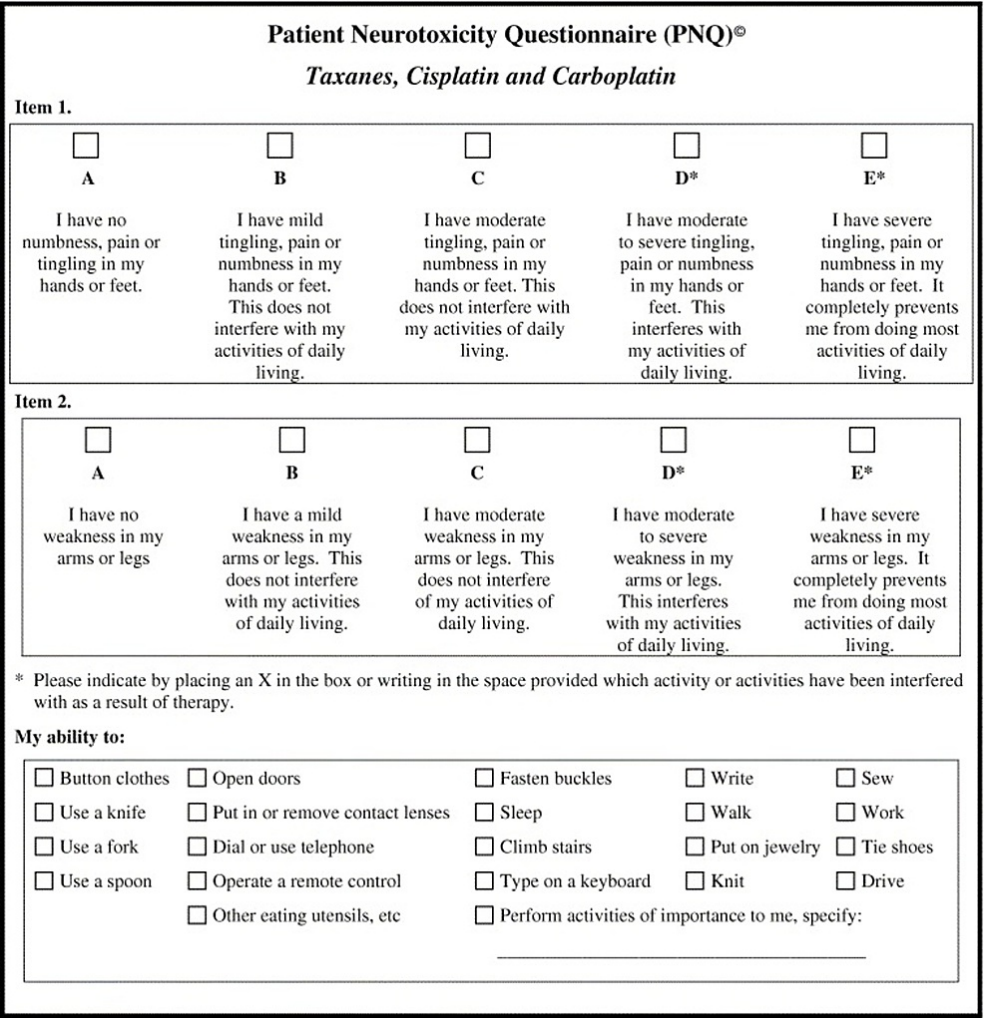
The original PNQ PNQ: Patient Neurotoxicity Questionnaire

**Figure 2:**
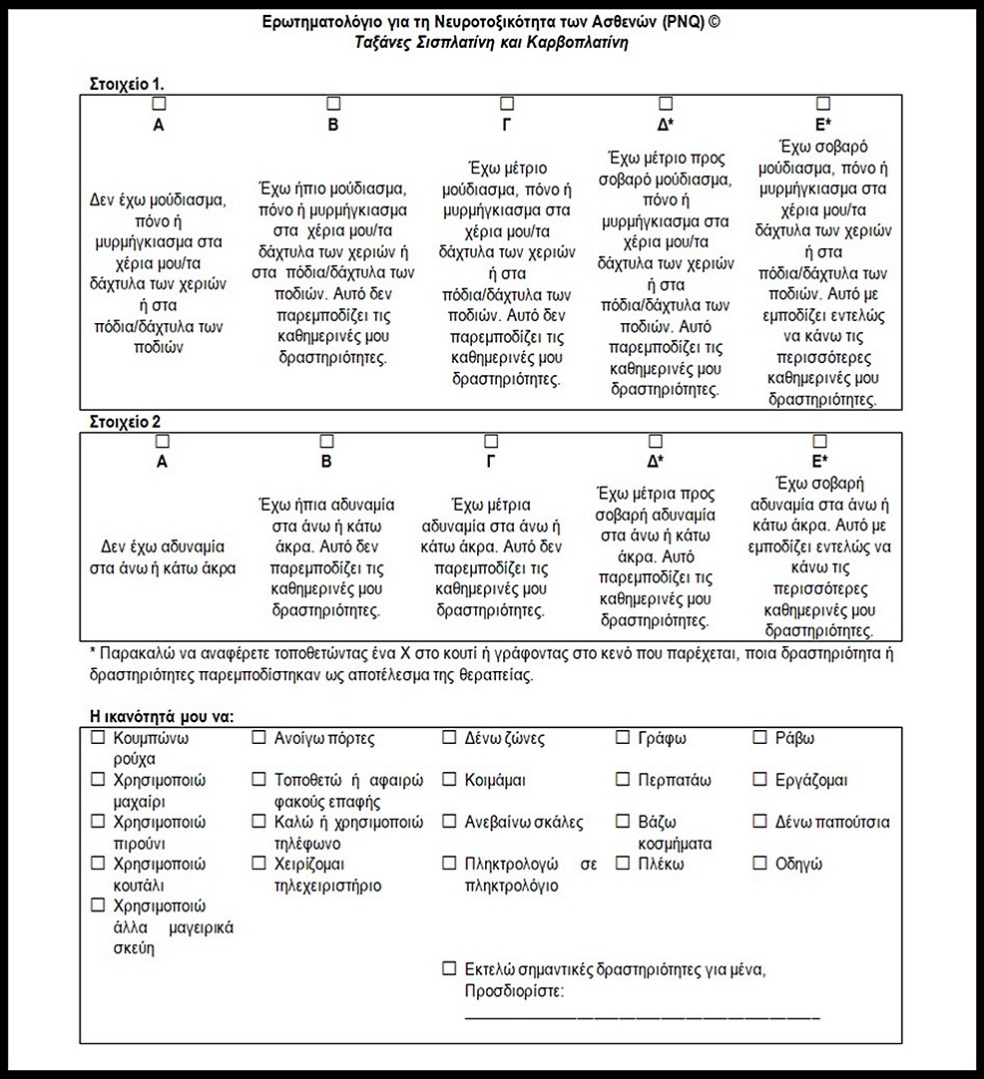
Greek version of the PNQ PNQ: Patient Neurotoxicity Questionnaire

The comparison of the newly translated texts highlighted the presence of numerous differences. Their nature consisted of scientific terminology, common terms, active and passive voice, synonyms, precise wording, and paraphrasing. Despite the different approaches of the two translators on the text, they both rendered the meaning of the original correctly. Thus, it was decided to prefer the terms that are closer to the original text and more comprehensive for the patient (Figures 1, 2).

The resulted text underwent a reversed translation by a third translator and was then compared to the original. After the comparison, there were many differences noted. Still, they were all judged both linguistically and conceptionally negligible as the original text’s meaning was maintained, and it was decided to proceed to the third validation stage.

Of the 100 patients, 46 were male and 54 female. The general age median was 65.8 while the male and female age median was 67.6 and 64.3. Most of the male patients (N=17) suffered from lung cancer, followed by colon (N=12), prostate (N=8), hepatic (N=5), stomach (N=2), and testicular (N=2) cancer patients. Most female patients had breast cancer (N=24), followed by lung cancer (N=14), colon (N=13), ovary (N=1), hepatic (N=1), and stomach cancer patients(N=1) (Tables 1, 2).

**Table 1:** Patients and cancer type (men, age median 67.6)

Cancer Type	Number	Percentage
Lung	17	36.9
Colon	12	26.1
Prostate	8	17.4
Hepatic	5	10.8
Testicular	2	4.3
Stomach	2	4.3

**Table 2:** Patients and cancer type (women, age median 64.3).

Cancer Type	Number	Percentage
Breast	24	44.4
Lung	14	25.9
Colon	13	24
Hepatic	1	1.9
Stomach	1	1.9
Ovary	1	1.9

Compliance at treatment's onset was 100% as all questionnaires were compiled. The compliance at the second, fourth, and sixth sessions was 98%, 95%, and 93%, respectively. The reasons were the deteriorated condition of the patient due to illness (N=2), declined condition of the patient due to adverse treatment effects (N=2), and absence of the physician responsible for the patient assessment (N=3). Of course, those were considered as missing data and were excluded from the next stages of the study.

A variation of Cohen's κ, the weighted κ, was applied. The latter considers not only the observed values but also the expected values based on agreement chances and the non-agreement weight coefficients that are usually equal to 1 [6,13-15].

At the distribution of both questionnaires' answers, the sensory part presented agreement regarding the A-C grades (0-2) and disagreement regarding the D-E (3-4) grades. A similar distribution is noted at the motor part with the agreement regarding the A-B (0-1) grades and disagreement at the C-E (2-4) grades. The Cohen's κ coefficients that arise are κ= 0.67 for the sensory part and κ= 0.58 for the motor part (Tables 3, 4).

**Table 3:** Correlation matrix between PNQ sensory and NCI-CTCAE for weighted Cohen's κ PNQ: Patient Neurotoxicity Questionnaire; NCI-CTCAE: National Cancer Institute-Common Terminology Criteria for Adverse Events

	NCI-CTCAE					
PNQ sensory		0	1	2	3	4
	A	101	0	0	0	0
	B	14	93	1	0	0
	C	0	41	75	1	0
	D	0	1	24	20	0
	E	0	0	0	10	5

**Table 4:** Correlation matrix between PNQ motor and NCI-CTCAE for weighted Cohen's κ PNQ: Patient Neurotoxicity Questionnaire; NCI-CTCAE: National Cancer Institute-Common Terminology Criteria for Adverse Events

	NCI-CTCAE					
PNQ motor		0	1	2	3	4
	A	121	3	0	0	0
	B	30	120	3	0	0
	C	1	41	37	0	0
	D	0	15	8	1	0
	E	0	0	1	3	1

Concerning the study of the validity, the answers to both questionnaires were distributed in increasing order. The Spearman's ρ that resulted was ρ=0.7 and ρ=0.98 for the sensory and motor part, respectively (Tables 5, 6).

**Table 5:** Spearman's ρ for PNQ sensory and NCI-CTCAE PNQ: Patient Neurotoxicity Questionnaire; NCI-CTCAE: National Cancer Institute-Common Terminology Criteria for Adverse Events

ρ=0,7	PNQ (Α-Ε)	Rank	NCI-CTCAE (0-4)	Rank
Α /0	101	3	114	4
Β/1	107	4	135	5
C/2	116	5	100	3
D/3	47	2	32	2
Ε/4	15	1	5	1

**Table 6:** Spearman's ρ for PNQ motor and NCI-CTCAE PNQ: Patient Neurotoxicity Questionnaire; NCI-CTCAE: National Cancer Institute-Common Terminology Criteria for Adverse Events

ρ=0,9	PNQ (Α-Ε)	Rank	NCI-CTCAE (0-4)	Rank
Α /0	125	4	153	4
Β/1	153	5	161	5
C/2	79	3	63	3
D/3	24	2	8	2
Ε/4	5	1	1	1

The resulting α coefficients for the PNQ were 0.59, 0.67, and 0.81 for the second, fourth, and sixth sessions. Regarding the NCI-CTCAE questionnaire, the corresponding α coefficients were 0.55, 0.57, and 0.75 [8,17,19-21].

**Figure 3:**
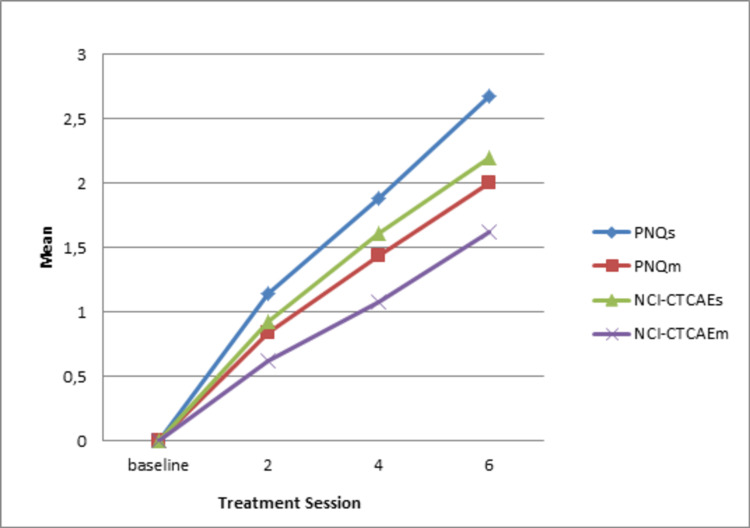
Mean values over time PNQ: Patient Neurotoxicity Questionnaire; NCI-CTCAE: National Cancer Institute-Common Terminology Criteria for Adverse Events

Regarding responsiveness and sensitivity, the symptoms' gravity was increased over time (P<0.0001) for both sensory and motor parts of the PNQ as expected marking CIPN worsening. Similar results were obtained with the NCI-CTCAE, thus with lower values (Table 7, Figure 3).

**Table 7:** Mean values over time for PNQ and NCI-CTCAE PNQ: Patient Neurotoxicity Questionnaire; NCI-CTCAE: National Cancer Institute-Common Terminology Criteria for Adverse Events

	Baseline		2ndSession		4th Session		6th Session			
	Mean	SD	Mean	SD	Mean	SD	Mean	SD	P-value	Cohen’s d
PNQ-sensory	0	0	1.13	0.37	1.89	0.61	2.7	0.75	<0.0001	2.6
PNQ-motor	0	0	0.82	0.51	1.44	0.61	2	0.86	<0.0001	1.7
NCI-CTCAE sensory	0	0	0.93	0.43	1.61	0.64	2.19	0.77	<0.0001	2
NCI-CTCAE motor	0	0	0.62	0.51	1.1	0.62	1.62	0.67	<0.0001	1.7

**Figure 4:**
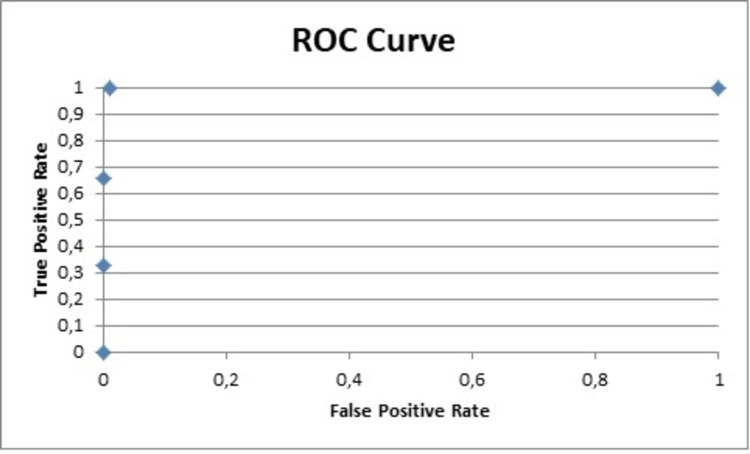
ROC curve for PNQ sensory PNQ: Patient Neurotoxicity Questionnaire; ROC: receiver operating characteristic

**Figure 5:**
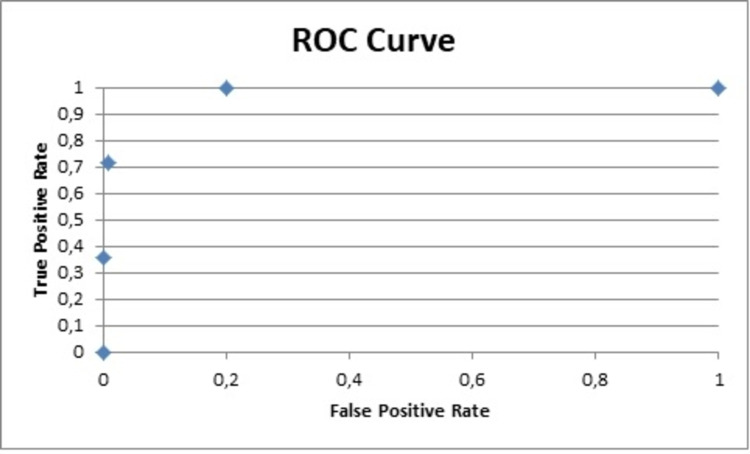
ROC curve for PNQ motor PNQ: Patient Neurotoxicity Questionnaire; ROC: receiver operating characteristic

**Figure 6:**
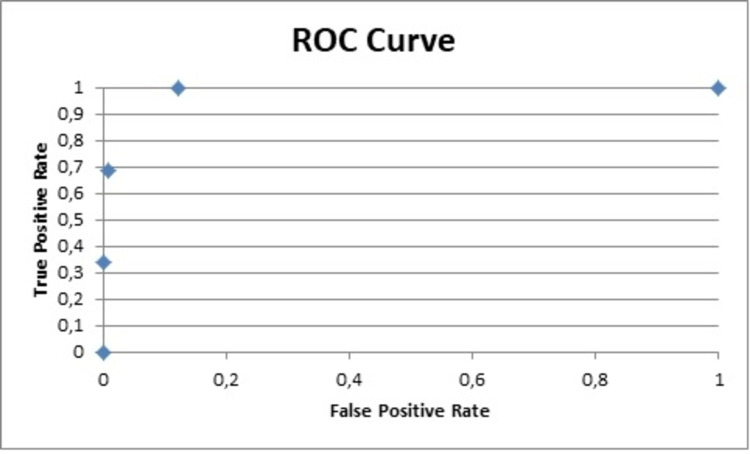
ROC curve for NCI-CTCAE sensory ROC: receiver operating characteristic; NCI-CTCAE: National Cancer Institute-Common Terminology Criteria for Adverse Events

**Figure 7:**
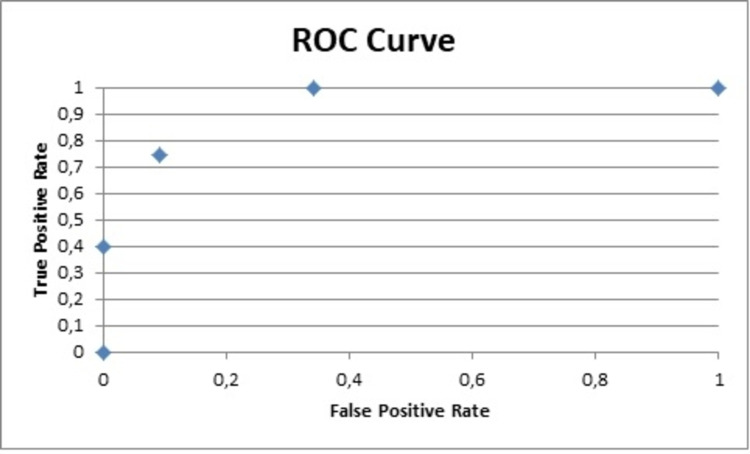
ROC curve for NCI-CTCAE motor ROC: receiver operating characteristic; NCI-CTCAE: National Cancer Institute-Common Terminology Criteria for Adverse Events

Other useful data obtained from the analysis was Cohen's d that reflects the importance of the results. The Cohen's d for the PNQ for the sensory and motor parts was 2.6 and 1.7, respectively, while for the NCI-CTCAE 2 and 1.6 [21]. The area under the curve (AUC) for the sensor and motor parts of the PNQ were 1 and 0.9, respectively, and 0.9 for both parts of the NCI-CTCAE (Figures 4-7) [9,17-20].

## Discussion

It is known that peripheral neuropathy affects the quality of life and should be carefully assessed clinically. Thus, when CIPN symptomatology is present, it is imperative to determine if it impacts the patients’ daily activities. The PNQ consists of targeted questions that are designed to extract specific information directly from the patient regarding the frequency as well as the severity of CIPN. The latter is executed by using a specific limit between “no affected” and “affected” everyday activities. The use of diagnostic methods for assessing CIPN has to be “handy and easy” for both patients and physicians. It should preferably not be interventional and finally to not require a great amount of time [4,6-12,22-26].

To apply a different diagnostic tool in the Greek clinical praxis, linguistic validation was imperative. Thus, when such a tool is translated from the original language to another, there is always a danger to miss the concept of the instrument itself. Thankfully, the PNQ has been shown to represent a simple questionnaire with content that could be rendered with clarity. That way, the Greek version remains close to the original, without the presence of dual interpretations and terms hard to conceive.

As mentioned above, a diagnostic tool should meet some specific criteria. The PNQ application presented a high compliance rate, with 92% of the patients compiling the questionnaire at the last treatment session and over 90% overall. That rate shows the easy application of the PNQ and highlights it as a tool of choice for assessing patients with CIPN-associated symptoms. In a similar study of Shimozuma et al., the PNQ again presented a compliance rate of over 90% again confirming the high rate of feasibility [1,7,12,14,16].

The analysis of the physician-centered NCI-CTCAE demonstrated that there is a tension of underestimating the importance of CIPN in quotidian activities in comparison to the patient-centered PNQ. Indeed, while the sensory section of both questionnaires presented an excellent concordance (κ=0.67), the motor section revealed a mediocre concordance (κ=0.57). That deviation between the two questionnaires is due to -and proves- the different point of view with which the patient's clinical condition is assessed. Indeed, the patients themselves evaluated their condition to be more aggravated in comparison to the observer physician a feat observed again in the study of Shimozuma et al. [7,14-16].

The PNQ validity was assessed with Spearman's ρ coefficient. The more that coefficient tends to 1, the greater the validity. The sensory part of the questionnaires presented a good association between variables (ρ=0.7), while the motor section presented an excellent one (ρ=0.98). In the study of Shimozuma et al., there was an excellent association between the sensory sections but a poor one regarding the motor sections. This could be due to the subjectivity of the symptoms (sensory or motor) from the part of the patient, in contrast to the observer physician who usually evaluates the patient strictly clinically. Another reason could be the cultural differences between the people of various countries [1,7,12,14,16].

At the last treatment session, the PNQ presented good reliability (α=0.81), more significant than that of the NCI-CTCAE (α=0.75). It has to be mentioned here that a limitation that could affect the result is that both questionnaires consist of just four questions (two for the sensory and two for the motor section). The Cronbach's α is usually used for questionnaires consisted of a higher number of questions [9,18-21].

The linear analysis of the data highlighted the ability of the PNQ to detect the CIPN effects on the patients over time, not only due to dosage increase but also due to the additive effects of chemotherapy medications. Those data could become the trigger of executing more specific studies (e.g., regarding the medication involved) to create protocols regarding the decision-making concerning chemotherapy and analgesic treatments. That analgesic intervention could regard not only acute pain but could also involve the creation of preventive treatment schemes. Those facts place the PNQ in a pivotal position as a data collector for CIPN [7,9,16,17].

The data analysis of the PNQ presented high AUC values for the sensory and motor part (1 and 0.9, respectively), which reflect an excellent specificity and highlight the value of the PNQ as an excellent diagnostic tool [9,18-21].

It has to be noted that a limitation of the present study is the heterogeneity of the sample. Nevertheless, this study represents the first approach involving the PNQ. Since the results produced are more than encouraging, it could be the stimulus for executing more specific studies (cancer type, sex, treatment). 

Another limitation is the lack of laboratory verification of neuropathy through the use of Electromyography. Nerve conduction studies are considered the “Golden Standard” in peripheral neuropathy diagnosis and should be applied in patients referring to CIPN symptoms. It has to be noted that to produce reliable results with this method, the patients should undergo Electromyography using the same device. Due to the colleagues' already increased workload and the psychological and physical stress of the patients under chemotherapy (many of whom had traveled from afar), it was decided that it was not prudent to burden them further [27].

## Conclusions

The PNQ represents an assessment tool that meets the aforementioned criteria. Plus it is fast and easy to compile, comprehensible, patient-centered, un-interventional, and it is unbound from the educational level of the patients. These facts render the PNQ easy to handle and offer to clinicians an accurate asset to use in order to manage the patients appropriately.

The PNQ validation in Greek adds a crucial diagnostic tool in the physicians' armory that can now draw the necessary information to modify the chemotherapy treatment schemes and organize an analgesic plan at both preventive and acute levels. Finally, it paves the way for researchers to examine a vast number of different situations from even greater samples but also samples with specific features. 
